# The molecular evolutionary characteristics of new isolated H9N2 AIV from East China and the function of vimentin on virus replication in MDCK cells

**DOI:** 10.1186/s12985-020-01351-9

**Published:** 2020-06-17

**Authors:** Yuan Nan Yu, Yang Zheng, Shan Shan Hao, Ze Zhang, Jia Xi Cai, Man Man Zong, Xiu Li Feng, Qing Tao Liu

**Affiliations:** 1 Key Laboratory of Animal Microbiology of China’s Ministry of Agriculture, College of Veterinary Medicine, Nanjing Agricultural University, Nanjing, 210095 China; 2 Institute of Veterinary Medicine, Jiangsu Academy of Agricultural Sciences, Nanjing, 210014 China; 3 MOE Joint International Research Laboratory of Animal Health and Food Safety, College of Veterinary Medicine, Nanjing Agricultural University, Nanjing, 210095 China

**Keywords:** H9N2 AIV, Phylogenetic characteristics, Molecular variations, Vimentin, siRNA, Virus replication

## Abstract

**Background:**

The low pathogenic H9N2 AIV caused the serious impact on the poultry industry and public safety. Our purpose was to investigate the molecular evolutionary characteristics of the new isolated H9N2 virus and investigate the intracellular target protein of H9N2 AIV replication in sensitive cells.

**Methods:**

AIV A/chicken/Shandong/LY1/2017 (H9N2) was isolated from the cloaca of the healthy chicken in Shandong, and the full-length eight gene segments of this isolated H9N2 AIV were amplified by RT-PCR and analyzed. MDCK cells were used as the target cell model, and VOPBA assay and LC-MS/MS were carried out to identify the virus-binding protein of H9N2 AIV. MDCK cells were pre-treated with the special antibody and siRNA, and treated with H9N2 AIV to detect the virus replication. Additionally, Vimentin-pcDNA3.0 was successfully constructed, and transinfected into MDCK cells, and then H9N2 AIV mRNA was detected with RT-PCR.

**Results:**

Phylogenetic analysis revealed that HA, NA, PB2, PB1, PA, NP and M seven genes of the isolated H9N2 AIV were derived from A/Chicken/Shanghai/F/98, while NS gene was derived from A/Duck/Hong Kong/Y439/97. The cleavage site sequence of HA gene of the isolated H9N2 AIV was a PARSSR G pattern, and the left side sequence (224 ~ 229) of receptor binding site was NGQQGR pattern, which were similar to that of A/Chicken/Shanghai/F/98. Following VOPBA assay, we found one protein of about 50KDa binding to H9N2 AIV, and the results of LC-MS/MS analysis proved that vimentin was the vital protein binding to H9N2 AIV. The pre-incubation of the specific antibody and siRNA decreased the viral RNA level in MDCK cells treated with H9N2 AIV. Furthermore, we found that over-expressed vimentin increased H9N2 AIV replication in MDCK cells.

**Conclusions:**

These findings suggested that the isolated H9N2 AIV might be a recent clinical common H9N2 strain, and vimentin protein might be one vital factor for H9N2 AIV replication in MDCK cells, which might be a novel target for design and development of antiviral drug.

## Background

H9N2 subtype avian influenza virus (AIV) has become responsible for the increasingly serious influence on poultry production and human health. Since 1994, H9N2 AIV was prevalent rapidly in many chicken farms and waterfowl populations, and became the most popular subtype of AIV in China [1–4]. The phylogenetic analysis of early isolates’ genes showed that H9N2 subtype had been circulating as a mainland China strain [5, 6]. Also, it was reported that the antigenicity of isolated H9N2 strains was different from that of vaccine strain in Guangdong, China [7]. Epidemiological studies showed that Neuraminidase (NA) gene of H7N9 influenza virus was homologous to that of H10N9 AIVs (A/chicken/Jiangsu/RD5/2013) isolated from the local live poultry market, whose internal genes were offered from the current popular H9N2 subtype AIV [8, 9]. Besides, H9N2 subtype AIV was the donor for the internal gene of the new H10N8 virus infected people [10, 11]. Similarly, some isolated H9N2 viruses shared human virus-like receptor specificity and substitution resembling human virus in the hemagglutinin (HA) site in Hong Kong [12, 13]. Pig introduced by H9 viruses would increase the risk of generating mammalian-adapted or reassorted variants, which might be potentially infectious to humans [14]. Therefore, it was important to investigate H9N2 AIV surveillance for the development of poultry industry and human safety.

Influenza viruses internalized and became into the early endosomal Endosomes (EEs) through the binding of HA protein with membrane surface receptor sites N-acetyl neuraminic acid (Neu Ac) and hydroxyacetyl neuraminic acid (Neu Gc), and then developed the late endosomal Endosomes (LEs) [15]. The viral genome was transported to the nucleus after recognition with the cell transporter, and the viral transcription and replication process was initiated [16]. The genetically similar H9N2 influenza A viruses presented the high or low pathogenicity in mice, in which multiple amino acid differences in PB2 gene may be responsible for the pathogenic difference of AIV for mice [17]. It has been reported that the variations of E627K and D701N in the PB2 gene might cause AIV through the species innate barrier to infect mammals, and the enhance virulence of the mutated AIV [18].

It was important to investigate AIV attachment to trachea in many avian species [19]. AIVs mainly attached to α2,3-linked SA, but also to combinations of α2,3- and α2,6-linked SA [20]. Kim found the differential influenza receptor expression pattern in mouse and human brains, and a disparity between influenza receptor distribution and regions with actual influenza infection [21]. To explore the possible intracellular receptor of AIV during virus infection and replication, in this paper, we employed the viral overlay protein binding assay to identify one receptor binding to H9N2 subtype AIV, and adopted the specific antibody block, siRNA and over-expression to study the effect of vimentin on H9N2 AIV replication in the sensitive cells.

## Methods and materials

### Virus, cells, and antibodies

H9N2 AIV used in this study was isolated from the cloaca of the healthy chicken in Shandong 2017, which was collected as samples of routinely ongoing surveillance. The hemagglutination inhibition with the special antibody confirmed that the isolate might be H9N2 subtype AIV. The virus was thrice propagated in 9-day-old specific-pathogen-free (SPF) embryonated chicken eggs, and then gene fragments were sequenced and comparatively analyzed. Madin-Darbycanine kidney (MDCK) cells were maintained in Dulbecco modified Eagle medium (DMEM) supplemented with 10% fetal bovine serum (FBS) and 5% CO_2_ at 37 °C.

Anti-vimentin monoclonal antibody was purchased from Abcam (ab45939) and anti-hemagglutinin polyclonal antibody was purchased from Jianchun Biotechnology (Nanjing). Anti- Glycerophosphate dehydrogenase (GAPDH) antibody (ab8245) was purchased from Abcam. Alkaline phosphatase conjugated goat anti-rabbit IgG and HRP-conjugated goat anti-mouse IgG secondary antibody was purchased from Univ Biotechnology (Shanghai). Vimentin siRNA was purchased from Santa Cruz (sc-156,015).

### RT-PCR

Based on the whole genome sequence of H9N2 AIV published in GenBank database of US National Center for Biotechnology Information (NCBI), showed in Table S1, the primer sequences of eight gene fragments of the H9N2 subtype AIV were designed, as shown in Table S2. Following Trizol instruction, the total RNAs were extracted from the allantoic fluids containing the isolated H9N2 AIV. According to the PrimeScriptTMRT Master Mix reverse transcription kit, cDNA was used as templates for polymerase chain reaction (PCR) amplification for eight genes fragments of the isolated virus.

### Gene sequencing and phylogenetic analysis

To eliminate the nucleotide acids error of eight genes in cDNA clones obtained using RT-PCR, five samples for each gene were sequenced by Nanjing Qing Ke biological company (Nanjing, China), and eight genes fragments were amplified. Also, MEGA5.3 was used to diagram the phylogenetic tree of each gene fragment [22], and to investigate the genetic evolution relationship between A/chicken/Shandong/LY1/2017 and other H9N2 strains.

### Viral overlay protein binding assay (VOPBA)

The samples of MDCK cells were obtained to extract all the intracellular proteins after ultrasonication and proteolysis. The collected proteins samples were analyzed with 12% SDS-PAGE, and transferred onto polyvinylidene fluoride (PVDF) membranes. After blocked, the transferred membranes were incubated with 1 multiplicity of infection (MOI) H9N2 subtype AIVs, and then incubated with rabbit polyclonal antibody special to HA protein of H9N2 AIV, and then incubated with goat anti-rabbit IgG secondary antibody. After screened, the results were developed with high resolution image acquisition system.

### Protein mass spectrometry sequencing

Simultaneously, the proteins samples were analyzed and stained with Coomassie staining, and the bands equivalent to the above western blot of the major virus binding band were recovered and analyzed for liquid chromatography tandem mass spectrometry (LC-MS/MS) analysis by Shanghai Zhongke Xinsheng Life Biotechnology Co., Ltd. (China, Shanghai). Simply, after reduction and alkylation, Trypsin was added into the examined samples to enzymatic hydrolysis for 20 h at 37 °C. After desalination and freeze-drying, the samples were dissolved in 0.1% FA solution, and then performed on Trap column for mass spectrometry. After MS2 scan, the raw files were obtained and searched in the related databases through Mascot 2.2 software. The detailed search parameters were showed in Table S3.

### Inhibition of H9N2 infection by Vimentin antibody

MDCK cells were incubated with 100 μg/mL anti-Vimentin antibody (ab45939) or rabbit IgG control at 37 °C for 2 h. After washing twice with phosphate buffer saline (PBS), the incubated MDCK cells were infected by 0.1 MOI H9N2 virus at 37 °C for 1.5 h, and cultured with DMEM for 36 h. Following Trizol instruction, the total RNAs of MDCK cells were extracted to be usaed as templates for RT-qPCR. The primers used for RT-qPCR were showed in Table S4. GAPDH was chosen as a internal gene control. The rabbit IgG incubated cells were used as a control for each comparison.

### RNA interference and H9N2 virus infection

MDCK cells in 12-well plate were transfected with vimentin siRNA and control siRNA according to Lipofectamine-3000 (Invitrogen) protocol. After 24 h, the transfected MDCK cells were infected with 0.1 MOI H9N2 AIV at 37 °C for 1 h, and were cultured with DMEM for 36 h, and then were collected to detect the vimentin mRNA and viral mRNA levels by RT-qPCR. Also, the knock-down expressed vimentin proteins were detected by western blot with vimentin antibody (ab45939) as the previous reported [23]. Control siRNA was used for control treatment.

### Over-expression of Vimentin and H9N2 virus infection

According to the published sequence NM_001287023.1 in Genbank database from NCBI, the primers of Vimentin were designed by Primer Premier 6.0, as showed in Table S5. Total RNA was extracted from MDCK cells by Trizol (Takara), and cDNA was synthesized by Reverse transcription Kit (Abm-Zhengjiang) to amplify vimentin gene. After sequenced, vimentin gene was ligated into pcDNA-3.0 vector using EcoR I and Hind III restriction sites to construct the eukaryotic expression vector vimentin-pcDNA3.0.

Vimentin-pcDNA3.0 was transfected by using Lipofectamine-3000 (Invitrogen) in 6-well plate, and vector pcDNA3.0 transfected MDCK cells were used as control. At 60 h post-transfection, vimentin mRNA in the transfected MDCK cells were detected by RT-qPCR, and the expression of vimentin proteins were checked by western blot with vimentin antibody (ab45939). Additionally, at 24 h post-transfection, the Vimentin-pcDNA3.0 transfected cells were incubated 0.1 MOI H9N2 AIVs for 1 h at 37 °C, and cultured in DMEM with 10% FBS for 36 h to detect H9N2 virus units by RT-qPCR.

### Statistical analysis

Results were illustrated in bar graphs as means ± standard deviation (SD) of three independent experiments. The statistical significances were analyzed by *t*-test or one-way ANOVA with significantly difference less than 0.05.

## Results

### Phylogenetic analysis of the isolated H9N2 AIV

To verify the detailed subtype,, the isolate were obtained after thrice propagated in 9-day-old SPF embryonated chicken eggs for comparative analysis of gene fragments. Eight genes fragments of AIV (A/chicken/Shandong/LY1/2017, abbreviated as LY1) were amplified, respectively, and the full length genome of LY1 was obtained (Table 1), which were available in GenBank accession numbers MH018674 - MH018681.

**Table 1:** Homology between the gene segments of LY1/ and that of some H9N2 isolates

LY1	Region of comparison (Nucleotide)	% Homology with H9N2 virus										
		JS7	JSC1	HBC1	F98	G9	BJ1	Y280	SD696	Wis66	Y439	G1
HA	57–1619	99.9	99.9	99.3	99.5	95.3	96.7	94.8	96.2	82.3	84.8	91.2
NA	20–1412	99.9	99.5	99.2	97.5	93.8	96	96.8	96.3	86.9	88.4	91.9
PB2	49–2284	99.6	99.7	99.4	98.8	86.3	92.4	91.6	92	83.9	92.5	86.1
PB1	25–2233	99.8	99.9	99.2	98.8	91.9	91.9	90.9	91.6	88.3	91.4	92.1
PA	53–2151	99.7	99.9	99.4	98.8	89.3	91.2	89.9	90.1	86.9	91.7	92.1
NP	56–1501	99.7	100.0	99.1	99	90.7	91.3	90.5	90.7	89	95	93.3
M	36–995	100.0	100.0	100.0	99.1	98.2	97.7	98.4	98.1	91.6	92.3	95.7
NS	41–851	99.9	100.0	99.4	90.4	90.8	91.6	90.8	91.2	92.1	96.4	90.6

Note: LY1: A/chicken/Shandong/LY1/2017; JS7: A/chicken/Jiangsu/7/2002; JSC1: A/swine/Jiangsu/C1/2008; HBC1: A/chicken/Hubei/C1/2007; F98: A/Chicken/Shanghai/F/98; G9:A/Chicken/Hong Kong/G9/97; BJ1: A/Chicken/Beijing/1/94; Y280:A/Duck/Hong Kong/Y280/97; SD696: A/Chicken/Shandong/6/96; Wis66:A/Turkey/Wisconsin/66; Y439: A/Duck/Hong Kong/Y439/97; G1: A/Duck/Hong Kong/Y439/97

**Fig. 1:**
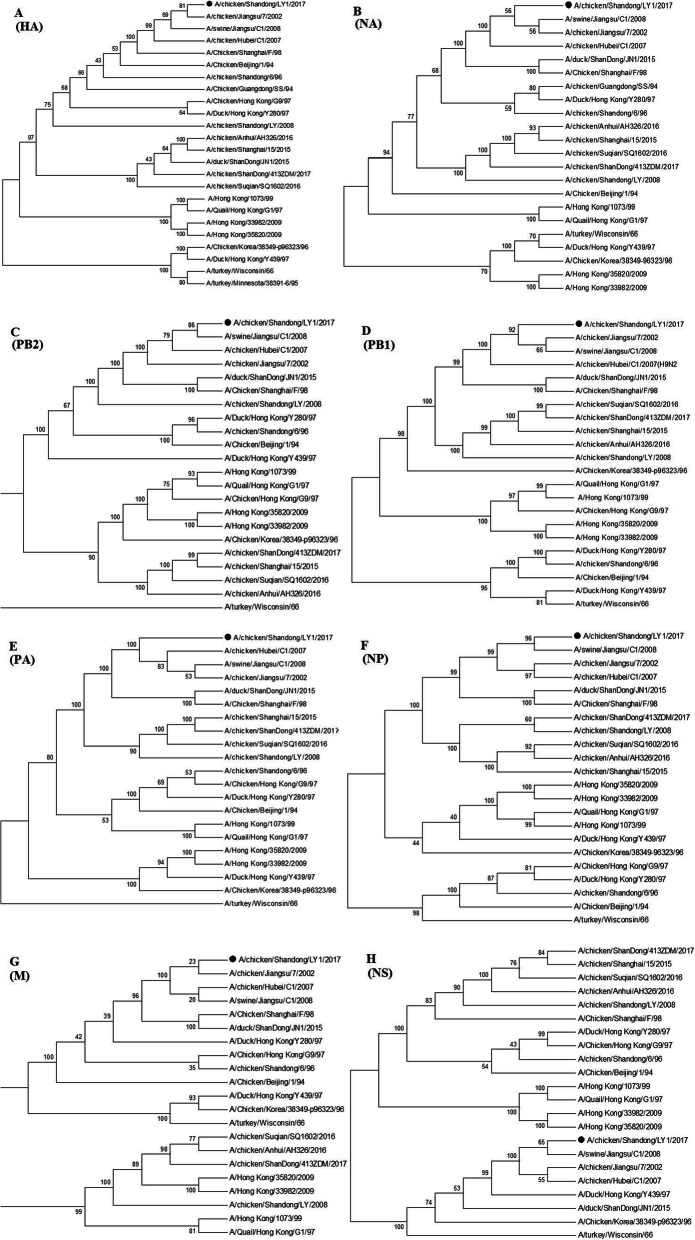
Phylogenetic trees for eight gene segments of LY1 (H9N2) virus. Nucleotides 57–1619 (1563 bp) of HA gene, 20–1412 (1393 bp) of NA gene, 49–2284 (2235 bp) of PB2 gene, 25–2233 (2209 bp) of PB1 gene, 53–2151 (2098 bp) of PA gene, 56–1501 (1445 bp) of NP gene, 36–995 (960 bp) of M gene and 41–851 (811 bp) of NS gene were analyzed. The trees were generated and edited with MEGE 5.0 software. Horizontal distances were proportional to the minimum number of nucleotide differences required to join nodes. All the sequences including LY1 strain can be found in Genbank (accession number available upon request)

To examine the molecular evolutionary relationship of this isolate virus, the phylogenetic comparisons of eight gene segments were carried out among LY1 and other H9N2 AIVs. The phylogenetic trees showed that HA, NA, PB2, PB1, PA, NP and M seven genes of LY1 were derived from A/Chicken/Shanghai/F/98 (F98)(Fig. 1a~g), whereas NS genes of the isolated virus shared a common ancestor with that of A/Duck/Hong Yong/Y439/97 virus (Y439) (Fig. 1h).

### Homology analysis of LY1 H9N2 AIV

It was noteworthy that the whole genome of LY1 had the highest homologies to that of the three isolates A/chicken/Jiangsu/7/2002 (JS7), A/swine/Jiangsu/C1/2008 (JSC1) and A/chicken/Hubei/C1/2007 (HBC1), in which homologies between eight full-length segments of LY1 and those of three viruses ranged from 99.1 to 100% (Table 1). Furthermore, the homologies in HA, NA, PB2, PB1, PA, NP and M seven genes between LY1 and F98 were from 97.5 to 99.5%, and NS gene between LY1 and Y439 was 96.4% (Table 1). These results suggested that LY1 might be a recent clinical common H9N2 strain.

### Molecular features of HA in LY1 strain

The cleavage site sequence of LY1 strain was a PARSSR↓G pattern, which was consistent with that of the Y280 subline (Clade h9.4.2, Table 2). Furthermore, three recently isolates of JS7, JSC1 and HBC1 and three referred strains of Y280, BJ1 and F98 strains were used as the reference strains to comparatively analyze the mutation of the receptor binding site of HA gene of LY1. The results showed that there no mutated amino acid in receptor binding sites of HA genes presented among LY1, JS7, JSC1 and HBC1 isolates (Table 3). The left side sequence (224 ~ 229) of receptor binding site of HA gene in LY1 was NGQQGR pattern, namely, Q at position 226, consistent with that of F98 and BJ1 strains. Besides, the amino acid at 190 site of HA gene in the isolate was V, which was same to that of BJ1 strain but different to that of F98 strain.

**Table 2:** Connecting-peptide at the cleavage site of H9N2 subtype AIV strains

Virus	Clade	Connecting-peptide at the cleavage site of HA
LY1	h9.4.2	PARSSR↓G
JS7	h9.4.2	PARSSR↓G
JSC1	h9.4.2	PARSSR↓G
HBC1	h9.4.2	PARSSR↓G
F98	h9.4.2	PARSSR↓G
G9	h9.4.2	PARSSR↓G
BJ1	h9.4.2	PARSSR↓G
Y280	h9.4.2	PARSSR↓G
SD696	h9.4.2	PARSSR↓G
Wis66	h9.1	PAVSSR↓G
Y439	h9.3	PAASNR↓G
G1	h9.4.1	PSRSSR↓G

Note: LY1: A/chicken/Shandong/LY1/2017; JS7: A/chicken/Jiangsu/7/2002; JSC1: A/swine/Jiangsu/C1/2008; HBC1: A/chicken/Hubei/C1/2007; F98: A/Chicken/Shanghai/F/98; G9:A/Chicken/Hong Kong/G9/97; BJ1: A/Chicken/Beijing/1/94; Y280:A/Duck/Hong Kong/Y280/97; SD696: A/Chicken/Shandong/6/96; Wis66:A/Turkey/Wisconsin/66; Y439:; G1: A/Duck/Hong Kong/Y439/97

**Table 3:** Analysis of Receptor Binding Sites in HA Gene in H9N2 AIVs

Strains	Left edge NGLQGR (224 ~ 229)	101 N	153 W	155 T	183 N	190 T	194 L	195 Y	Left edge GTSKA (138 ~ 142)
Y280	+	+	+	+	+	+	+	+	+
BJ1	NGQQGR	+	+	+	+	V	+	+	+
F98	NGQQGR	+	+	+	+	A	+	+	+
JS7	NGQQGR	+	+	+	+	V	+	+	+
JSC1	NGQQGR	+	+	+	+	V	+	+	+
HBC1	NGQQGR	+	+	+	+	V	+	+	+
LY1	NGQQGR	+	+	+	+	V	+	+	+

Note: LY1: A/chicken/Shandong/LY1/2017; Y280:A/Duck/Hong Kong/Y280/97; BJ1: A/Chicken/Beijing/1/94; F98: A/Chicken/Shanghai/F/98; JS7: A/chicken/Jiangsu/7/2002; JSC1: A/swine/Jiangsu/C1/2008; HBC1: A/chicken/Hubei/C1/2007. “+”: With the same amino acid sites

The potential glycosylation sites analysis on the HA of LY1 showed that LY1 had seven potential glycosylation sites at the same positions as those of three recently isolates, which were less than that of three typical strains (Table 4). Compared with the three typical strains Y280, BJ1 and F98, the amino acid at position 218 of HA gene in LY1 was T, resulting in the glycosylation site deletion at position 218. Furthermore, we found that the amino acid at position 145 of HA gene of LY1 had mutated to be N, resulting in a new glycosylation site NGT at this position.

**Table 4:** Prediction of the potential glycosylation sites of HA protein in H9N2 AIVs

Site Sequence Strains	29 NST	141 NVS	145 NGT	218 NRT	298 NTT	305 NVS	313 NCS	492 NGT	551 NGS
Y280	+	+	–	+	+	+	–	+	+
BJ1	+	NVT	–	+	+	+	–	+	+
F98	+	+	–	+	+	+	–	+	+
JS7	+	+	+	–	+	+	–	+	+
JSC1	+	+	+	–	+	+	–	+	+
HBC1	+	+	+	–	+	+	–	+	+
LY1	+	+	+	–	+	+	–	+	+

Note:LY1: A/chicken/Shandong/LY1/2017; Y280:A/Duck/Hong Kong/Y280/97; BJ1: A/Chicken/Beijing/1/94; F98: A/Chicken/Shanghai/F/98; JS7: A/chicken/Jiangsu/7/2002; JSC1: A/swine/Jiangsu/C1/2008; HBC1: A/chicken/Hubei/C1/2007. “+”: With the same glycosylation sites; “-”: Without glycosylation sites

### Molecular characteristics of NA in LY1 strain

It was observed that the deletions in the stalk and potential N-glycosylation sites of NA gene in LY1 were same to that of F98, JS7, JSC1 and HBC1 strain (Table 5). Compared to that of BJ1, the deletion of three amino acids ITE at position 62 to 64 in NA gene leaded to the deletion of glycosylation sites at position 61 in NA protein of LY1 strain. Unexpectedly, one amino acid mutant from NST to NNT at position 69 were occurred in NA protein of three recently isolates JS7, JSC1 and HBC1, which did not change the glycosylation sites at position 69 in NA protein of these three isolates.

**Table 5:** Amino acid sequence analysis of NA gene of AIV strains

Site Sequence	Deletion in the stalk	Potential N-glycosylation sites							Hemadsorbing sites		
	62 ~ 64	61	69	86	146	200	234	402	367 ~ 372	400 ~ 403	431 ~ 433
	ITE	NIT	NST	NWS	NGT	NAT	NGT	NWS	KKDSRS	SDNW	PQE
Strains											
Y280	+	–	+	+	+	+	+	+	KEDSRS	+	+
BJ1	–	+	+	+	+	+	+	+	+	+	+
F98	+	–	+	+	+	+	+	+	KKDSRS	+	+
JS7	+	–	NNT	+	+	+	+	+	KVDSRS	+	+
JSC1	+	–	NNT	+	+	+	+	+	KVDSRS	+	+
HBC1	+	–	NNT	+	+	+	+	+	KVDSRS	+	+
LY1	+	–	+	+	+	+	+	+	KVDSRS	+	+

Note:LY1: A/chicken/Shandong/LY1/2017; Y280:A/Duck/Hong Kong/Y280/97; BJ1: A/Chicken/Beijing/1/94; F98: A/Chicken/Shanghai/F/98; JS7: A/chicken/Jiangsu/7/2002; JSC1: A/swine/Jiangsu/C1/2008; HBC1: A/chicken/Hubei/C1/2007. “+”: With the same amino acid sequence; “-”: Without the same amino acid sequence

The receptor binding sites of NA protein in LY1 mainly existed at three positions, 367 KVDSRS 372, 400 SDNW 403 and 431 PQE 433. It was found that the KVDSRS pattern in NA protein of LY1, which was same to that of the three recently strains JS7, JSC1 and HBC1, and different to that of three typical strains F98, Y280 and BJ1 strains (Table 5).

### Identification of Vimentin as H9N2 AIV binding protein

**Fig. 2:**
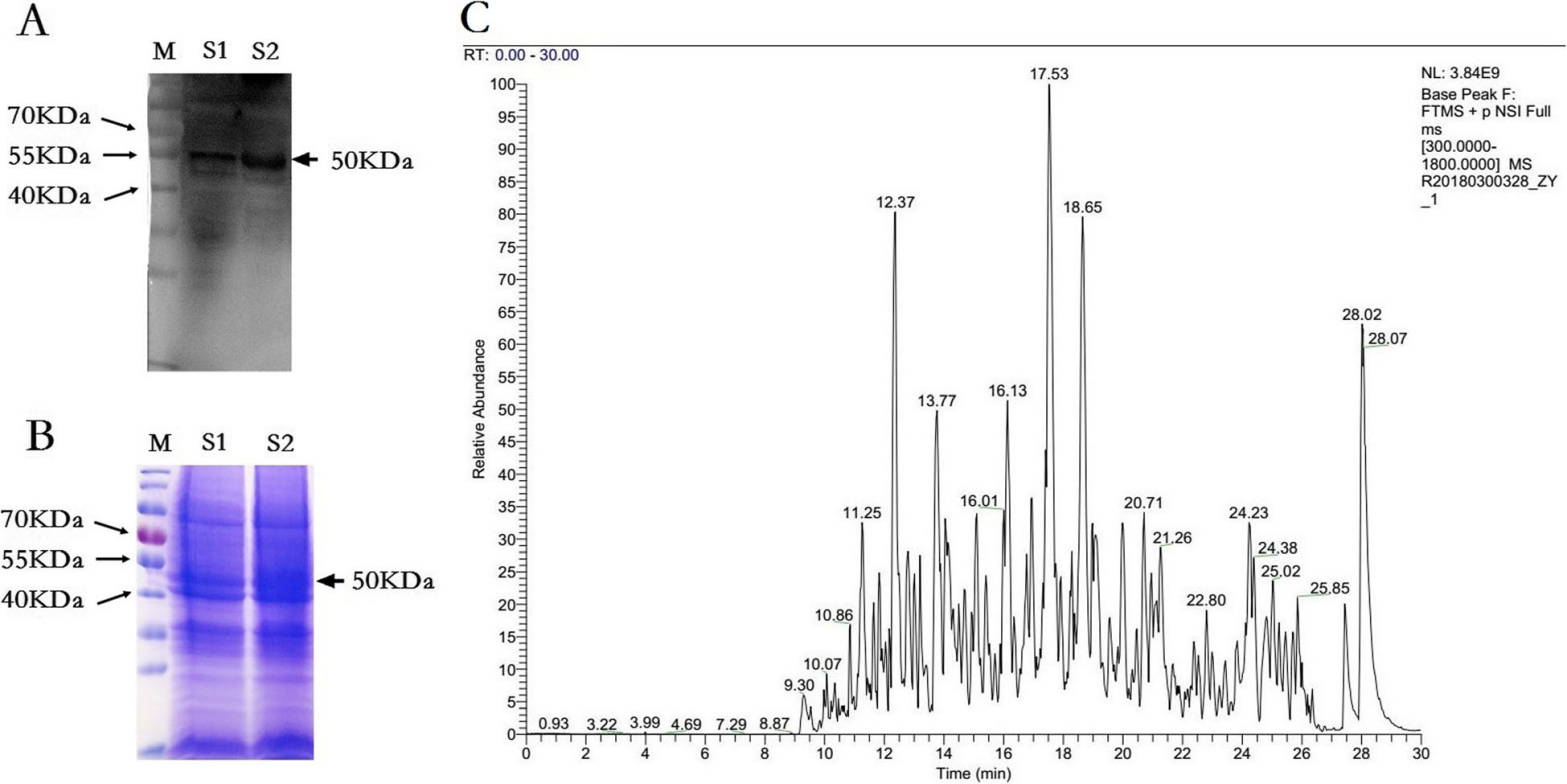
Strategy for identification vimentin protein as H9N2 virus-binding membrane protein. **a** Detection membrane protein isolated from MDCK cells binding with H9N2 virus by VOPBA. **b** The equivalent coomassie staining band for LC-MS/MS. M: Low molecular weight protein marker; S1: Parallel sample 1; S2: Parallel sample 2. **c** The LC-MS/MS Mass spectrum for the coomassie staining band

To identify the protein in MDCK cells involved in H9N2 virus attachment, we employed VOPBA with polyclonal antibody against HA protein of H9N2 virus, and found one strong binding-band with molecular mass about 50KDa, in which the same bands appeared in the both parallel samples (Fig. 2a). Also, the equivalent protein bands with about 50KDa from duplicated coomassie blue gel was obtained to be analyzed for the binding protein by biotechnology company (Fig. 2b). The LC-MS/MS analysis spectrum for the band protein was showed in Fig. 2c. According to the value of UniquePepCount and molecular mass in the related databases through Mascot 2.2 software, the molecular weight of vimentin was 53.5KDa, which was consistent with that of binding-band protein band, and the UniquePepCount of vimentin was 27 (Table 6), suggesting vimentin might be the protein binding to H9N2 AIV in MDCK cells.

**Table 6:** The receptor protein binding protein sequencing analysis

	UniquePepCount	PepCount Sequence	Cover Percent	Molecular Weight	Reference
Vimentin	27	179	55.15%	53,596.98	tr|F1PLS4|F1PLS4_CANLF Vimentin OS=Canis lupus familiaris OX = 9615 GN=VIM PE = 3 SV = 1

### The antibody against Vimentin inhibited H9N2 virus entry

**Fig. 3:**
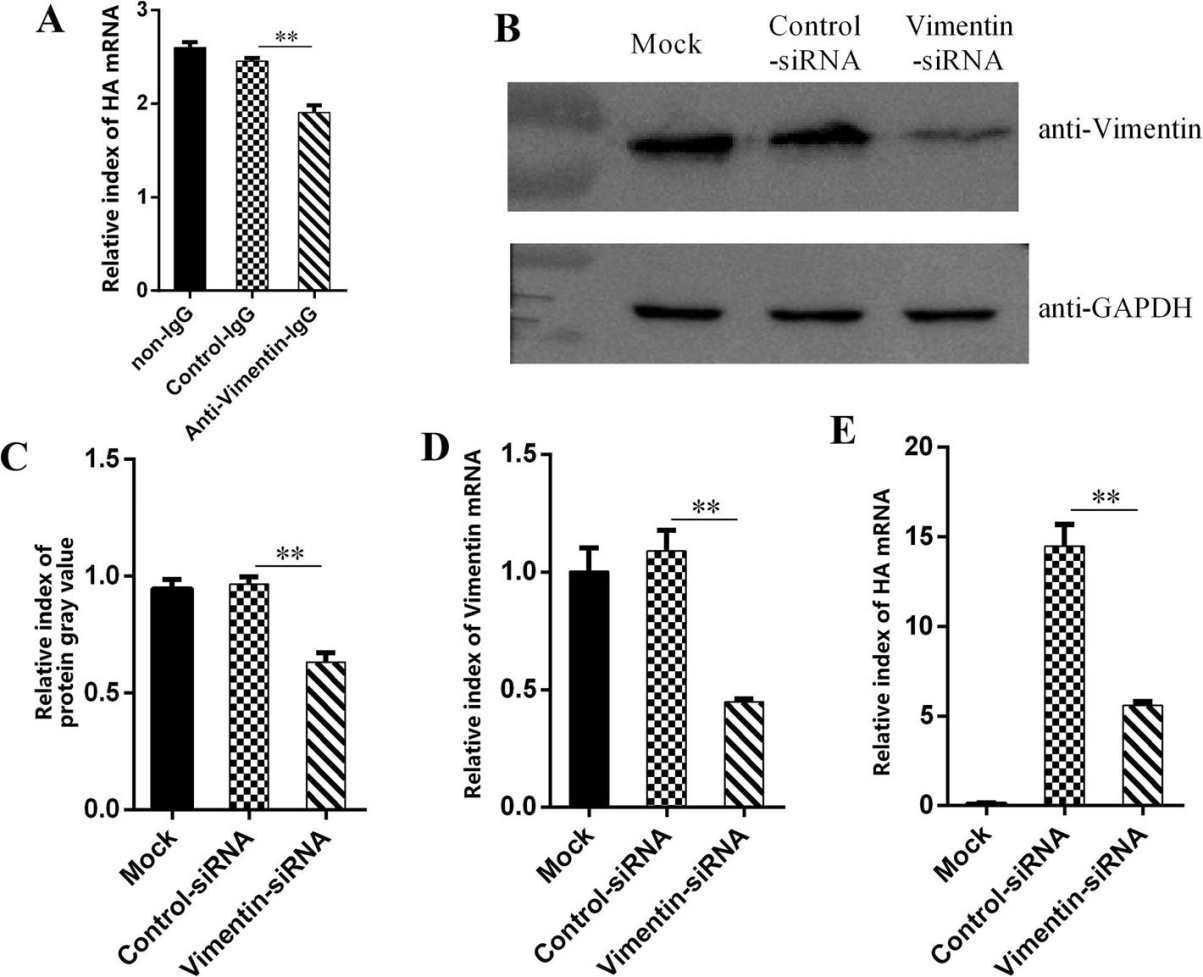
Antibody and siRNA special to vimentin decreased H9N2 virus replication in MDCK cells. **a** Vimentin antibody inhibits H9N2 virus replication in MDCK cells. **b** siRNA decreased the expressions of vimentin by western blot. **c** Grayscale analysis of the expressions of vimentin (**b**). **d** siRNA decreased the expressions of vimentin by qPCR . **e** H9N2 virus replication in MDCK cells with or without siRNA treatment. **, *p* < 0.01

To determine the role of membrane vimentin protein in H9N2 virus entry, MDCK cells were preincubated with anti-vimentin antibody, and then infected with H9N2 AIV. The qPCR results showed that the viral RNA level was significant lower in anti-vimentin antibody treated cells than that of IgG control (Fig. 3a). It was demonstrated that anti-vimentin antibody might a binding restriction with membrane during H9N2 virus entry.

### Small interference RNA (siRNA) of Vimentin reduced H9N2 virus replication

To further detect the function of vimentin in H9N2 virus reproduction, MDCK cells were pretreated with siRNA specific to vimentin, and then infected with H9N2 AIV for 36 h. The results showed that the vimentin expressions were decreased in siRNA treated cells, compared with that of control (Fig. 3b), whose protein expressions were analyzed with grayscale analysis (Fig. 3c). Compared to control-siRNA, the protein level of vimentin was reduced to 60% in siRNA treated cells. Also, the qPCR results showed that vimentin mRNA levels in siRNA treated cells were significantly lower than that of control-siRNA (Fig. 3d), which were reduced to 40%.

The qPCR results showed that the viral RNA levels of siRNA-transinfected group cells were 5.55, while control-siRNA cells were 14.45, in which H9N2 virus replication in siRNA-transinfected group were significantly decreased, compared to that of control-siRNA (Fig. 3e). These results indicated that vimentin protein had the important role in H9N2 virus replication.

### Over-expressed Vimentin in MDCK cells improved H9N2 virus replication

**Fig. 4:**
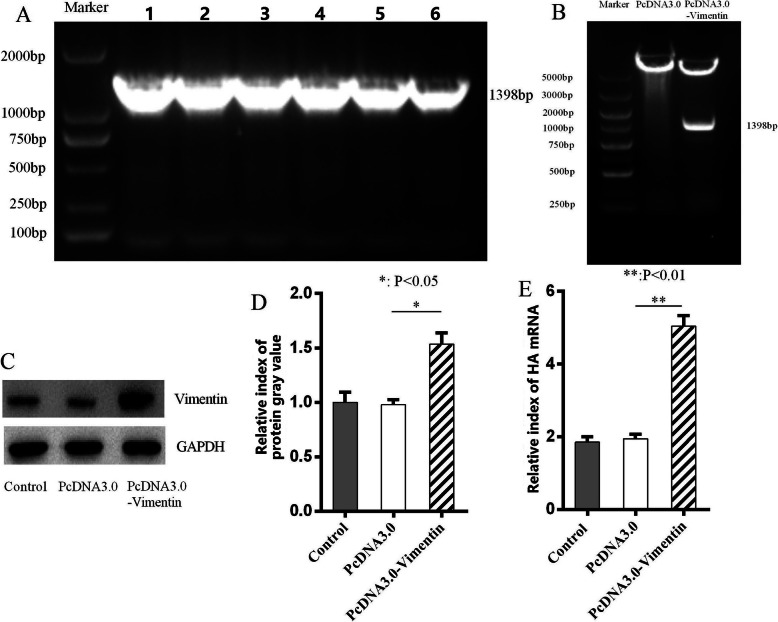
Over-expression of vimentin enhanced H9N2 virus replication. **a** Vimentin gene was cloned by PCR; 1 ~ 6: PCR sample 1 ~ 6. **b** The enzyme digestion of the positive recombinant plasmid pcDNA3.0-Vimentin. **c** Over-expressed vimentin protein analyzed by western blot in MDCK cells transinfected with plasmid pcDNA3.0-Vimentin. **d** Grayscale analysis of over-expressed vimentin protein (**b**). **e** H9N2 virus RNA replication detected by RT-qPCR. *, *p* < 0.05, and **, *p* < 0.01

The 1398 bp vimentin gene was amplified (Fig. 4a), and was inserted into the vector pcDNA-3.0 to construct the pcDNA 3.0-vimentin plasmid, which was confirmed with restriction enzyme identification (Fig. 4b). Also, compared with that of pcDNA-3.0 vector controls, the expressions of vimentin protein in MDCK cells transinfected with pcDNA 3.0-vimentin plasmid were significantly increased (Fig. 4c), and the mRNA levels of vimentin in pcDNA 3.0-vimentin-transinfected MDCK cells were significantly increased (Fig. 4d). Furthermore, the viral RNA levels in MDCK cells transinfected with vimentin-pcDNA3.0 were significantly higher than that of pcDNA-3.0 vector controls (Fig. 4e). These results suggested that the over-expression of vimentin in MDCK cells might stimulate H9N2 virus replication.

## Discussion

. Epidemiological investigation showed H9N2 subtype avian influenza virus might be the donor for the internal genes of H7N9 [8, 9] and H10N8 [10, 11]. Therefore, monitoring on molecular characteristics of H9N2 subtype AIV would be important in prevention and control on avian influenza, which play the vital role on poultry industry and human health [24]. An innovative subline naming system for AIVs was proposed, and lineages and sublineages were classified according to genetic distances, topology of the phylogenetic trees and distributions of the viruses in hosts, regions and time, including of four h9.1, h9.3, h9.4.1 and h9.4.2 subline [5, 22, 25]. In this paper, the homologies of eight gene segments of LY1 with the recent three isolates JS7, JSC1 and HBC1 suggested that LY1 might be a recent clinical common H9N2 strain. F98 and Y439 were the representative strains of H9N2 avian influenza virus belong to different subline [22, 25]. Furthermore, the NS gene of LY1 was homologous to Y439 strain (belongs to h9.3 subline), whereas other seven genes of LY1 were homologous to F98 strain (belongs to h9.4.2 subline). These results suggested that LY1 might have a potential evolutionary relationship with h9.3 and h9.4.2 subline AIV strains.

The connecting peptide of HA of LY1 was PARSSR↓G motif, a characteristic of H9N2 viruses of land-based poultry [8]. In this paper, there were the same receptor binding sites and seven glycosylation sites in HA genes presented among LY1, JS7, JSC1 and HBC1 isolates. Generally, the quantity of the glycosylation sites on HA of H9 viruses might be associated to the derived species, including of duck, quail and chicken [26]. The effect of these mutations of the some sites on the function of HA protein remained to be explained.

It was found that there were same hemadsorbing sites of the NA gene between that of JS7, JSC1, HBC1, and LY1, which were different from that of F98, and Y280. Unexpectedly, one amino acid mutant from NST to NNT were occurred in NA protein of JS7, JSC1, HBC1, but did not present in LY1. This finding suggested that the potential biological significance of this molecular marker in LY1 isolate remained to be elucidated.

The mechanism of influenza virus entry and replication in cells needs to be further investigated. In this paper, employing VOPBA and mass spectroscopy analysis, we found the vimentin might be the potential binding proteins to LY1 strain in MDCK cells. It has been reported that vimentin protein was associated with multiple cellular functions, and was required for parvoviral infection [27]. Also, vimentin protein was related with pH-dependent infection of parvovirus, dengue virus replication and release [28, 29]. To investigate the effect of vimentin on H9N2 AIV replication, in this research, MDCK cells were treated with preincubated with anti-vimentin antibody, and then infected with H9N2 AIV. The results hinted that anti-vimentin antibody might has a restriction on H9N2 AIV binding and entry into MDCK cells. Also, we observed that when vimentin was knocked down with siRNA, the viral RNA levels of MDCK cells were significantly decreased, whereas the viral RNA levels of MDCK cells were significantly increased in MDCK cells with over-expressed vimentin. It was reported that vimentin was important for Epstein-Barr Virus LMP1-mediated Akt and ERK activation and transformation of rodent fibroblasts [30]. Cellular vimentin is also a specific host binding partner for 2C of FMDV [31], and Vimentin rearrangement plays a structural role in anchoring DENV2 to replication sites [32], and in facilitating efficient viral RNA replication with NS4A Protein [33], and in enhancing PRRSV growth by interacting with ANXA2 [34]. These results suggested that vimentin as intermediate filament in MDCK cells should be an important intracellular molecule for H9N2 virus entry and replication. Given influenza virus A may use multiple receptors for cell entry, such as N-acetylneuraminic acid and glycolyl neuraminic acid receptors, vimentin might be used as a transport of viral vRNP during AIV replication. However, the interactions mechanism between H9N2 virus and vimentin needed to be the further research.

## Conclusions

In summary, the isolated H9N2 AIV might be a recent clinical common H9N2 strain, and vimentin was identified as the binding protein to H9N2 AIV, and the results of the specific antibody, siRNA and over-expression proved that vimentin protein was one vital factor for H9N2 virus replication in MDCK cells, which might be a novel target for antiviral drug design and development.

## Supplementary information

**Additional file 1: Table S1.** The reference strains of H9N2 subtype influenza virus and their abbreviations.

**Additional file 2: Table S2.** Specific primers for eight gene segments of LY1 strain.

**Additional file 3: Table S3.** The detailed search parameters of mass analysis.

**Additional file 4: Table S4.** The qPCR primers for HA gene of H9N2 Virus and Vimentin.

**Additional file 5: Table S5.** The PCR primers of Vimentin for construction of vimentin-pcDNA3.0.

## Data Availability

The datasets used and analyzed during the current study are included within this article and in additional file.

## Abbreviations

AIV: Avian influenza virus
NA: Neuraminidase
HA: Hemagglutinin
EEs: Early endosomal Endosomes
Les: Late endosomal Endosomes
vRNPs: Viral ribonucleoprotein complexes
SPF: Specific pathogen free
MDCK: Madin-Darbycanine kidney
DMEM: Dulbecco modified Eagle medium
FBS: Fetal bovine serum
GAPDH: Glycerophosphate dehydrogenase
NCBI: National Center for Biotechnology Information
PCR: Polymerase chain reaction
RT-PCR: Reverse transcription polymerase chain reaction
VOPBA: Viral Overlay Protein Binding Assay
PAGE: Polyacrylamide gel electrophoresis
PVDF: Polyvinylidene fluoride
MOI: Multiplicityof infection
LC-MS/MS: Liquid chromatography tandem mass spectrometry
PBS: Phosphate buffer saline
SD: Standard deviation
