# Supplementary material for: The molecular evolutionary characteristics of new isolated H9N2 AIV from East China and the function of vimentin on virus replication in MDCK cells
Source: Virol J. 2020 Jun 17;17:78. doi: 10.1186/s12985-020-01351-9 (PMC7302367; doi:10.1186/s12985-020-01351-9)
Supplement: Supplementary file 1 — Additional file 1: Table S1. The reference strains of H9N2 subtype influenza virus and their abbreviations. [file 12985_2020_1351_MOESM1_ESM.docx]

**Table S1. The reference strains of H9N2 subtype influenza virus and their abbreviations**

| Virus strain | Abbreviation |
| --- | --- |
| A/chicken/Beijing/1/94 | BJ1 |
| A/chicken/Hong Kong/G9/97 | G9 |
| A/chicken/Korea/38349-p96323/96 | Kr96323 |
| A/chicken/Shandong/6/96 | SD696 |
| A/chicken/Shanghai/F/98 | F98 |
| A/Duck/Hong Kong/Y280/97 | Y280 |
| A/Duck/Hong Kong/Y439/97 | Y439 |
| A/Hong Kong/1073/99 | 1073 |
| A/Hong Kong/33982/2009 | 33982 |
| A/Hong Kong/35820/2009 | 35820 |
| A/Quail/Hong Kong/G1/97 | G1 |
| A/turkey/Wisconsin/1966 | Wis66 |
| A/chicken/Jiangsu/7/2002 | JS7 |
| A/swine/Jiangsu/C1/2008 | JSC1 |
| A/chicken/Hubei/C1/2007 | HBC1 |
