# Supplementary material for: The molecular evolutionary characteristics of new isolated H9N2 AIV from East China and the function of vimentin on virus replication in MDCK cells
Source: Virol J. 2020 Jun 17;17:78. doi: 10.1186/s12985-020-01351-9 (PMC7302367; doi:10.1186/s12985-020-01351-9)
Supplement: Supplementary file 2 — Additional file 2: Table S2. Specific primers for eight gene segments of LY1 strain. [file 12985_2020_1351_MOESM2_ESM.docx]

**Table S2. Specific primers for eight gene segments of LY1 strain**

| Gene |  | Upstream primers （5’-3’） | Downstream primers （5’-3’） | Product (bp) |
| --- | --- | --- | --- | --- |
| HA |  | AGCAAAAGCAGGGGAATT | AGTAGAAACAAGGGTGTTTTTGC | 1742 |
| NA |  | AGCAAAAGCAGGAGTGAAAATGA | GGCAAGTAGAAACAAGGAGTT | 1458 |
| PB2 | Fragment 1 (1～1425) | AGCGAAAGCAGGTCAAATATAT | CTCTGGTGGAGCTGCTGCAAATGGT | 2341 |
|  | Fragment 2 (1094～2341) | AGGCATTTCCAAAAGGAATGCAAAG | AGTAGAAACAAGGGTGTTTTTGC |  |
| PB1 | Fragment 1 (1～1269) | AGCAAAAGCAGGCAAACC | GAGGTCTTATTTTCTCGATTTTCT | 2341 |
|  | Fragment 2 (1090～2341) | AGAAAATCGAGAAAATAAGACCTC | ACTCCTGCTTGTATTCCCTCA |  |
| PA | Fragment 1 (1～1295) | AGCGAAAGCAGGTACTGATCC | ACTCCCTTCATTATGTATTC | 2233 |
|  | Fragment 2 (1088～2233) | AACAAGCCAATTGAAGTGGGCACT | AGTAGAAACAAGGTACTTTTTT |  |
| NP | Fragment 1 ( 1～1244) | AGCAAAAGCAGGGTA | CCTCTGTTGGTTGGTGTT | 1565 |
|  | Fragment 2 ( 740～1565) | TTCCAAACAGCAGCACA | AGTAGAAACAAGGGTATTTTT |  |
| M |  | AGCAAAAGCAGGTAG | AGTAGAAACAAGGTAGTTTTT | 1027 |
| NS |  | AGCAAAAGCAGGGTG | AGTAGAAACAAGGGTGTTTT | 890 |
