# Supplementary material for: The molecular evolutionary characteristics of new isolated H9N2 AIV from East China and the function of vimentin on virus replication in MDCK cells
Source: Virol J. 2020 Jun 17;17:78. doi: 10.1186/s12985-020-01351-9 (PMC7302367; doi:10.1186/s12985-020-01351-9)
Supplement: Supplementary file 3 — Additional file 3: Table S3. The detailed search parameters of mass analysis. [file 12985_2020_1351_MOESM3_ESM.doc]

**Table S3**. The detailed search parameters of mass analysis

| Option | Parameter |
| --- | --- |
| Enzyme | Trypsin |
| Database | Uniport_dog_29583_20180409.fasta |
| Fixed modifications | Carbamidomethyl (C) |
| Variable modifications | Oxidation (M) |
| Missed Cleavage | 2 |
| Peptide Mass Tolerance | 20ppm |
| Fragment Mass Tolerance | 0.1Da |
| Filter by score>=20 | |
