# Supplementary material for: The molecular evolutionary characteristics of new isolated H9N2 AIV from East China and the function of vimentin on virus replication in MDCK cells
Source: Virol J. 2020 Jun 17;17:78. doi: 10.1186/s12985-020-01351-9 (PMC7302367; doi:10.1186/s12985-020-01351-9)
Supplement: Supplementary file 4 — Additional file 4: Table S4. The qPCR primers for HA gene of H9N2 Virus and Vimentin. [file 12985_2020_1351_MOESM4_ESM.doc]

**Table S4**. The qPCR primers for HA gene of H9N2 Virus and Vimentin

| Primers name | Sequence from 5’ to 3’ | Product size |
| --- | --- | --- |
| GAPDH-F | TGTCCCCACCAATGT | 156bp |
| GAPDH-R | TCCGATGCCTGCTTCACTACT |
| HA-F | TTACCCTGTTCAAGACGCCC | 139bp |
| HA-R | GCCACACTCGTTGTTGTGTC |
| Vimentin-F | GAGGCTGCTAACCGGAACAA | 152bp |
| Vimentin-R | ATTAGTCCCTTTGAGTGCATCC |
