# Supplementary material for: The molecular evolutionary characteristics of new isolated H9N2 AIV from East China and the function of vimentin on virus replication in MDCK cells
Source: Virol J. 2020 Jun 17;17:78. doi: 10.1186/s12985-020-01351-9 (PMC7302367; doi:10.1186/s12985-020-01351-9)
Supplement: Supplementary file 5 — Additional file 5: Table S5. The PCR primers of Vimentin for construction of vimentin-pcDNA3.0. [file 12985_2020_1351_MOESM5_ESM.doc]

**Table S5**. The PCR primers of Vimentin for construction of vimentin-pcDNA3.0

| Primers name | Sequence from 5’ to 3’ | Product size |
| --- | --- | --- |
| Vimentin-F | CCCAAGCTTGGATGTCCACCAGGTCTGTGT | 1398bp |
| Vimentin-R | CCGGAATTCTCACTCAAGGTCATCATGATG |

Note: EcoR I and Hind III restriction sites were added in sequence with underlined.
